# Passive Smoking and Breast Cancer Risk among Non-Smoking Women: A Case-Control Study in China

**DOI:** 10.1371/journal.pone.0125894

**Published:** 2015-04-27

**Authors:** Bin Li, Lian Wang, Min-Shan Lu, Xiong-Fei Mo, Fang-Yu Lin, Suzanne C. Ho, Cai-Xia Zhang

**Affiliations:** 1 Department of Medical Statistics and Epidemiology, School of Public Health, Sun Yat-sen University, Guangzhou, China; 2 Epidemiology Research Unit, the First Affiliated Hospital of Sun Yat-sen University, Guangzhou, China; 3 Department of Vascular Surgery, the First Affiliated Hospital of Sun Yat-sen University, Guangzhou, China; 4 Nursing Department, the First Affiliated Hospital of Sun Yat-sen University, Guangzhou, China; 5 Division of Epidemiology, The Jockey Club School of Public Health and Primary Care, The Chinese University of Hong Kong, Hong Kong SAR, China; Kagoshima University Graduate School of Medical and Dental Sciences, JAPAN

## Abstract

**Background:**

The role of passive smoking on breast cancer risk was unclear. This study aimed to evaluate the association between passive smoking and breast cancer risk among Chinese women.

**Methods/Principal Findings:**

A hospital-based case-control study, including 877 breast cancer cases and 890 controls, frequency-matched by age and residence, was conducted. A structured questionnaire was used to collect information on passive smoking history through face-to-face interview by trained interviewers. Unconditional logistic regression models were used to estimate the association between passive smoking and breast cancer risk. A positive association between any passive smoking exposure and breast cancer risk was observed. Compared with women who were never exposed to passive smoking, women who were ever exposed had a higher breast cancer risk, with the adjusted odds ratio (OR) and 95% confidence interval (CI) of 1.35 (1.11-1.65). Similar result was found on home passive smoking exposure and breast cancer risk, but not on workplace passive smoking exposure. Women who were ever exposed to tobacco smoke at home had a higher risk of breast cancer compared with never exposed women, with the adjusted OR (95% CI) of 1.30 (1.05-1.61). Home passive smoking exposure showed significant dose-response relationships with breast cancer risk in smoker-years, cigarettes/day and total pack-years (*P*_trend_=0.003, 0.006 and 0.009, respectively). An increased total smoker-years of any passive exposure significantly elevated the risk of breast cancer (*P*_trend_<0.001). Positive associations and dose-response relationships were found among postmenopausal women and all subtypes of estrogen receptor (ER) and progesterone receptor (PR) status of breast cancer.

**Conclusions:**

Passive smoking was associated with an increased risk of breast cancer among non-smoking Chinese women. A stronger positive association with breast cancer risk was seen mainly among postmenopausal women.

## Introduction

Many studies have examined the association between tobacco smoking and breast cancer risk [1–9]. However, the findings have been controversial. Some studies have reported no increased risk [4, 10–13], while others have reported increased risk for passive smoking exposure [1, 9, 14–19]. The review by Canadian expert panel showed that the evidence for a relationship between passive smoking and breast cancer remained tenuous, although they suggested that the relationship between passive smoking and breast cancer in younger, primarily premenopausal women was consistent with causality [20]. The most recent review suggested that the role of passive smoking was less clear [21].

In China, traditionally, few women are smokers, but the rate of passive smoking has known to be high. A survey conducted between 2005 and 2007 showed a high rate of 43.6% [22]. Even higher level of passive smoking was reported in younger women and in rural areas [22–24]. Some studies have evaluated the association between passive smoking and breast cancer risk among Chinese women [14, 19, 25–28]. Although most studies [14, 25–28] reported the positive association of passive smoking with breast cancer risk, these studies had relatively smaller sample size with 108 to 704 study subjects [14, 25, 26], and few studies have used quantitative measures to evaluate the exposures of passive smoking both at home and in the workplace [19]. Inadequate evaluations of exposure may result in an under-estimation of the risks, if they do exist [20]. We conducted this case-control study in Guangdong Province, China to investigate the association of passive smoking at home and in the workplace with breast cancer risk.

## Materials and Methods

### Ethics Statement

The procedures and protocols of the study were approved by The Ethical Committee of School of Public Health, Sun Yat-sen University. All participants signed informed consent forms before the interview.

### Study Subjects

Breast cancer cases and controls were recruited through two stages. The first stage was conducted from June 2007 to August 2008 and the second stage was from September 2011 to September 2013. Potential case patients were recruited from inpatients admitted to the surgical units of two affiliated hospitals of Sun Yat-sen University. Eligible cases were female subjects with histologically confirmed breast cancer diagnosed no more than 3 months before the interview, aged 25–70 years and natives of Guangdong province or having lived in Guangdong for at least 5 years. Women were excluded if they had a history of breast cancer or other cancers. Totally, 925 eligible cases were identified and 896 were interviewed, with a response rate of 96.9%.

Control subjects were patients admitted to the same hospitals during the same time period as the case subjects. Eligibility criteria for controls were the same as described for the cases except that they had no history of any cancers. They were frequency matched with cases by age (5-year interval) and residence (rural/urban). These patients presented with a wide spectrum of non-neoplastic conditions including eye disorders (glaucoma, uveitis, keratitis, pterygium, dacryocystitis, and optic neuritis), ear-nose-throat diseases (sudden deafness, acute bacterial/viral otitis media, sinusitis, deviation of nasal septum, tonsillitis), trifacial neuralgia, varicose veins, osteoarthritis, degenerate joint disease, orthopedics diseases, facial paralysis and acute appendicitis. In total, 939 controls were identified and 912 were interviewed, with 2.9% patients refused to participate.

### Data Collection

A structured questionnaire was used to collect information through face-to-face interview by trained interviewers. The collected information included socio-demographic and anthropometric parameters, dietary habits, menstrual and reproductive factors, use of hormone and contraceptive drugs, family history of cancer, alcohol drinking, active smoking, passive smoking history, disease history, and physical activity. The interview time was limited to exposures that occurred before diagnosis date for cases and the interview date for controls. Relevant medical information, medical diagnosis, and histological findings were abstracted from the hospital medical records.

Women were classified as non-smokers if they reported never smoking or smoking less than 100 cigarettes over their lifetime [29]. Passive smoking history was collected for two exposure sources. First, the subject was asked whether her husband or other family members ever smoked in her house, then she was asked the average number of cigarettes they smoked per day and the number of years she had been exposed at home. Second, the subject was asked whether someone ever smoked within three meters around her in her workplace, then she was asked the number of people and the number of years she had been exposed in the workplace. Women were categorized as having been exposed to passive smoking if they reported ever being exposed to tobacco smoke at home or in the workplace. Among women who were passive smokers, the duration or intensity of exposure with smoker-years, cigarettes/day or pack-years of exposure were calculated. Smoker-years were defined as sum of the number of years of exposure to each smoker [15]. Total smoker-years were calculated as the sum of smoker-years at home and in the workplace. Pack-years were defined as the number of years of exposure multiplied by the pack of cigarettes (1 pack = 20 cigarettes) smoked per day for a given smoker [10]. Pack-years were summed across smokers to generate a total pack-years measure.

Body mass index (BMI) was calculated by dividing weight (kg) by height (m^2^). Menopausal status was defined as at least 12 months since the last menstrual cycle. Women were considered to be premenopausal if they were currently menstruating, or if they were not menstruating because of a hysterectomy and younger than 50 years old. Women were defined as postmenopausal if they had either undergone a natural menopause, or surgery to remove both ovaries, or if their ovarian function was unknown but they were older than 50 years [30].

### Statistical analysis

Since the socio-demographic and established breast cancer risk factors of the two-stage study subjects are comparable, we pooled the two stage data for these analyses. The analysis excluded 41 subjects (19 cases and 22 controls) who reported past or current history of personal tobacco smoking. Analyses were based on the remaining 1767 non-smokers (877 cases and 890 controls).

Differences in characteristics were assessed by using either *χ* ^*2*^ tests for categorical variables or t tests for continuous variables. Unconditional logistic regression models were used to estimate odds ratios (OR) and 95% confidence intervals (CI) for the association between passive smoking and breast cancer risk. Based on the comparison of baseline characteristics between cases and controls, the following variables, BMI, physical activity, age at menarche, age at first live birth, age at menopause, history of benign breast disease, and mother/sister/ daughter with breast cancer, were selected to be adjusted for as potential confounding factors. Age, residence and study stage were also controlled for in all logistic models. Tests for trend were performed by entering categorical variables as continuous parameters in the models. Stratified analyses by menopausal status were conducted. As breast cancer is a heterogeneous disease with estrogen receptor (ER) and progesterone receptor (PR) subtypes, stratified analyses by ER/PR status were also conducted. All analyses were performed using SPSS 13.0 (SPSS Inc., Chicago, Illinois, USA). All tests were two-sided, with *P* values < 0.05 indicating statistical significance.

## Results

Compared to controls, cases were more likely to have higher BMI, an earlier age at menarche, a later age at first live birth, a later age at menopause, a history of benign breast disease and a family history of breast cancer, and were less likely to be physically active (Table 1). All of the above variables were considered potential confounders and adjusted for in subsequent analyses. No significant differences were found between cases and controls in socio-demographic factors, including marital status, educational level, occupation, and household income, or in reproductive factors, including nulliparous, number of live births, months of breast feeding, menopausal status, and use of an oral contraceptive.

**Table 1:** Comparison of cases and controls by selected socio-demographic characteristics.

Characteristics	Cases (n = 877)	Controls (n = 890)	*P*-value
Age (mean ± SD)	46.77 ± 9.95	46.65 ± 10.25	0.808
Residence (n, %)			0.887
Rural	404 (46.1)	407 (45.7)	
Urban	473 (53.9)	483 (54.3)	
Marital status (n, %)			0.568
Married	819 (93.4)	837 (94.0)	
Unmarried/divorced/widowed	58 (6.6)	53 (6.0)	
Educational level (n, %)			0.283
Primary school or below	213 (24.3)	252 (28.3)	
Junior high school	240 (27.4)	225 (25.3)	
Senior high school/secondary technical school	212 (24.2)	207 (23.3)	
College or above	212 (24.1)	206 (23.1)	
Occupation (n, %)			0.052
Administrator/other white collar worker	368 (42.0)	344 (38.7)	
Blue collar worker	174 (19.8)	219 (24.6)	
Farmer/other	334 (38.2)	327 (36.7)	
Income (yuan/mo.) (n, %)			0.226
≤ 2000	176 (20.1)	188 (21.1)	
2001–5000	305 (34.8)	271 (30.4)	
5001–8000	217 (24.7)	225 (25.3)	
≥ 8001	179 (20.4)	206 (23.2)	
Body mass index, BMI (mean ± SD)	22.91 ± 3.35	22.50 ± 3.13	0.008
Regular drinker (n, %)	31 (3.5)	29 (3.3)	0.748
Physical activity (exercise for health; n, %)			0.002
Never	360 (41.0)	322 (36.2)	
Occasionally	129 (14.7)	102 (11.5)	
≥1 time/wk.	388 (44.3)	466 (52.3)	
Age at menarche, yr. (mean ± SD)	14.70 ± 1.85	14.90 ± 1.84	0.027
Nulliparous (n, %)	49 (5.6)	50 (5.6)	0.978
Age at first live birth^a^ (yr.) (mean ± SD)	25.76 ± 3.56	25.34 ± 3.39	0.012
Number of live births^a^ (mean ± SD)	1.94 ± 1.10	2.01 ± 0.04	0.248
Months of breast feeding^b^ (mean ± SD)	22.92 ± 19.23	22.85 ± 18.87	0.944
Age at menopause^c^ (yr.) (mean ± SD)	49.57 ± 4.00	48.85 ± 3.94	0.029
Menopausal status (n, %)			0.523
Premenopausal	591 (67.4)	587 (66.0)	
Postmenopausal	286 (32.6)	303 (34.0)	
Mother/sister/daughter with breast cancer (n, %)	33 (3.8)	10 (1.1)	<0.001
Ever had benign breast disease (n, %)	357 (40.7)	207 (23.3)	<0.001
Ever used an oral contraceptive (n, %)	47 (5.4)	38 (4.3)	0.285

Abbreviation: SD = standard deviation; BMI = body mass index; mo. = month; wk. = week; yr. = year.

^a^Among women who have had a live birth.

^b^Among women who have breast fed.

^c^Among menopausal women.

As shown in Table 2, of all subjects, 495 (56.4%) cases and 442 (49.7%) controls reported ever having been exposed to passive smoking at home or in the workplace. Compared with women who were never exposed to passive smoking, women who were ever exposed had a higher risk of breast cancer, with the adjusted OR (95% CI) of 1.35 (1.11–1.65). When subjects were categorized according to sources of exposure, 39.7% of cases and 37.4% of controls were exposed only at home, 7.1% of cases and 7.2% of controls were exposed only in the workplace, and 9.7% of cases and 5.1% of controls reported both exposures. The adjusted ORs (95% CIs) of breast cancer were 1.30 (1.05–1.61) for passive smoking exposure only at home, 1.05 (0.71–1.56) for passive smoking exposure only in the workplace and 2.17 (1.45–3.23) for both exposures, compared with women unexposed to passive smoking.

**Table 2:** Overall associations between passive smoking and breast cancer risk.

	Cases (N = 877)		Controls (N = 890)				
	Freq.	%	Freq.	%	OR^a^	95% CI	*P*-value
Passive smoking exposure							
Never exposed	382	43.6	448	50.3	1.00		
Ever exposed^b^	495	56.4	442	49.7	1.35	1.11–1.65	0.002
Passive smoking categories							
None	382	43.5	448	50.3	1.00		
Home only	348	39.7	333	37.4	1.30	1.05–1.61	0.016
Workplace only	62	7.1	64	7.2	1.05	0.71–1.56	0.791
Home and workplace	85	9.7	45	5.1	2.17	1.45–3.23	<0.001

Abbreviation: Freq. = frequency; OR = odds ratio; CI = confidence interval.

^a ^All ORs and 95% CIs were calculated in a logistic regression model adjusted for age, residence, study stage, BMI, physical activity, age at menarche, age at first live birth, age at menopause, mother/sister/daughter with breast cancer and history of benign breast disease.

^b^ Passive smoking exposure at home or in the workplace.

Passive smoking exposure at home was examined in detail. Women who were ever exposed to passive smoking at home (53.1% cases and 45.8% controls) had a higher risk of breast cancer compared with women who were never exposed to passive smoking, with the adjusted OR (95% CI) of 1.30 (1.05–1.61). Dose-response relationships between breast cancer risk and smoker-years, cigarettes per day and pack-years of exposure at home were observed (*P*_trend_ = 0.003, 0.006 and 0.009, respectively). Compared with women with no passive smoking exposure, the adjusted ORs (95% CIs) of more than 26 smoker-years, more than 16 cigarettes per day and more than 16 pack-years of exposure at home were 1.66 (1.21–2.26), 1.56 (1.17–2.09) and 1.61 (1.17–2.19), respectively (Table 3).

**Table 3:** Overall association between passive smoking exposure at home and breast cancer risk.

	Cases (N = 815)^a^		Controls (N = 826)					
	Freq.	%	Freq.	%	OR ^b^	95% CI	*P*-value	*P*_trend_-value
Passive smoking exposure at home								
Never exposed	382	46.9	448	54.2	1.00			
Ever exposed	433	53.1	378	45.8	1.30	1.05–1.61	0.015	
Smoker-years of exposure at home ^c^								0.003
Never exposed	382	48.6	448	55.9	1.00			
1–15	112	14.3	112	14.0	1.06	0.76–1.46	0.741	
16–25	137	17.4	123	15.3	1.18	0.87–1.60	0.282	
> = 26	155	19.7	119	14.8	1.66	1.21–2.26	0.001	
Cigarettes/day smoked by family at home^d^								0.006
Never exposed	382	47.4	448	55.3	1.00			
1–4	140	17.4	119	14.7	1.33	0.99–1.79	0.061	
5–15	115	14.3	116	14.3	1.10	0.80–1.50	0.569	
> = 16	169	20.9	127	15.7	1.56	1.17–2.09	0.002	
Total pack-years of exposure at home^e^								0.009
Never exposed	382	48.9	448	56.6	1.00			
1–4	148	18.9	124	15.6	1.30	0.96–1.75	0.085	
5–15	110	14.1	114	14.4	1.06	0.77–1.46	0.698	
≥16	141	18.1	106	13.4	1.61	1.17–2.19	0.003	

Abbreviation: Freq. = frequency; OR = odds ratio; CI = confidence interval.

^a^ Excluded all women who reported only passive smoke exposure in the workplace, remained 815 cases and 826 controls.

^b^ ORs adjusted for age, residence, study stage, BMI, physical activity, age at menarche, age at first live birth, age at menopause, mother/sister/daughter with breast cancer, history of benign breast disease and workplace passive smoking exposure.

^c^ 53 women (29 cases, 24 controls) who reported no information on smoker-years were excluded.

^d^ 25 women (9 cases, 16 controls) who reported no information on cigarettes/day smoked by family were excluded.

^e^ 68 women (34 cases, 34 controls) whose pack-years were not calculated due to missing information were excluded.

Analysis on the association between passive smoking exposure at workplace and breast cancer risk (418 cases and 465 controls) (Table 4) showed no association, with the adjusted OR (95% CI) of 1.19 (0.80–1.78) comparing women who were ever exposed to tobacco smoke in the workplace with never exposed women. No significant association was found between smoker-years of exposure in the workplace and breast cancer risk (*P*_trend_ = 0.313).

**Table 4:** Overall association between passive smoking exposure in the workplace and breast cancer risk.

	Cases (N = 418)^a^		Controls (N = 465)					*P*_trend_-value
	Freq.	%	Freq.	%	OR^b^	95% CI	*P*-value	
Passive smoking exposure in the workplace								
Never exposed	271	64.8	356	76.6	1.00			
Ever exposed	147	35.2	109	23.4	1.19	0.80–1.78	0.397	
Smoker-years of exposure in the workplace^c^								0.313
Never exposed	271	66.7	356	78.4	1.00			
1–15	50	12.3	40	8.8	1.21	0.71–2.06	0.491	
16–35	40	9.9	23	5.1	1.46	0.79–2.69	0.225	
≥ 36	45	11.1	35	7.7	1.23	0.67–2.25	0.497	

Abbreviation: Freq. = frequency; OR = odds ratio; CI = confidence interval.

^a^ Among 883 women (418 cases, 465controls) who had ever been employed and excluding all women who reported only passive smoke exposure at home.

^b^ ORs adjusted for age, residence, study stage, BMI, physical activity, age at menarche, age at first live birth, age at menopause, mother/sister/daughter with breast cancer, history of benign breast disease and passive smoking at home.

^c^ 23 women (12 cases, 11 controls) who reported no information on smoker-years were excluded.

A strong dose-response relationship and a positive association were observed between total smoker-years of passive smoking exposure at home and in the workplace and breast cancer risk. Compared with women who were never exposed to passive smoking, the adjusted ORs (95% CIs) was 0.95 (0.70–1.29) for 1–16 smoker-years, 1.34 (1.04–1.74) for 17–30 smoker-years, and 1.95 (1.43–2.66) for more than 31 smoker-years, respectively (*P*_trend_ < 0.001).

Analyses stratified by menopausal status showed a positive association between passive smoking and breast cancer risk, primarily among postmenopausal women. Compared with non-exposed women, the adjusted OR (95% CI) was 1.83 (1.29–2.60) for women with any passive smoking exposure and 1.80 (1.24–2.61) for women ever exposure to passive smoking at home. The significant dose-response relationships between breast cancer risk and smoker-years, cigarettes per day and total pack-years of exposure at home were also found only among postmenopausal women (*P*_trend_ = 0.001, < 0.001 and 0.001, respectively). But the significant dose-response relationship between total smoker-years of any exposure and breast cancer risk was found in both pre/post-menopausal women. No significant association was found between passive smoking in the workplace and breast cancer risk in both the pre/post-menopausal women (Table 5).

**Table 5:** Association between passive smoking exposure and breast cancer risk by menopausal status.

	Premenopausal								Postmenopausal							
	Cases (N = 591)		Controls (N = 587)				*P*- value	*P*_trend_- value	Cases (N = 286)		Controls (N = 303)				*P*- value	*P*_trend_- value
	Freq.	%	Freq.	%	OR	95% CI			Freq.	%	Freq.	%	OR	95% CI		
Passive smoking exposure ^a^																
Never exposed	270	45.7	289	49.2	1.00				112	39.2	159	52.5	1.00			
Ever exposed^b^	321	54.3	298	50.8	1.18	0.93–1.50	0.176		174	60.8	144	45.5	1.83	1.29–2.60	0.001	
Total smoker-years of any exposure ^a^ ^,^ ^c^								0.034								<0.001
Never exposed	270	47.6	289	51.4	1.00				112	41.6	159	54.3	1.00			
1–16	99	17.5	108	19.2	1.01	0.72–1.41	0.974		14	5.2	26	8.9	0.70	0.33–1.46	0.339	
17–30	133	23.4	122	21.7	1.18	0.86–1.62	0.318		61	22.7	52	17.7	1.77	1.10–2.85	0.019	
≥ 31	65	11.5	43	7.7	1.68	1.08–2.61	0.021		82	30.5	56	19.1	2.32	1.48–3.64	<0.001	
Passive smoking exposure at home^d^ ^,^ ^c^																
Never exposed	270	49.7	289	53.8	1.00				112	41.2	159	55.0	1.00			
Ever exposed	273	50.3	248	46.2	1.10	0.84–1.43	0.477		160	58.8	130	45.0	1.80	1.24–2.61	0.002	
Smoker-years of exposure at home^d^ ^,^ ^c^								0.452								0.001
Never exposed	270	51.1	289	55.5	1.00				112	43.6	159	56.6	1.00			
1–15	98	18.5	91	17.4	1.09	0.75–1.57	0.654		14	5.4	21	7.5	0.87	0.40–1.88	0.721	
16–25	116	21.9	102	19.6	1.08	0.77–1.54	0.645		21	8.2	21	7.5	1.54	0.76–3.09	0.230	
≥ 26	45	8.5	39	7.5	1.20	0.72–1.95	0.493		110	42.8	80	28.4	2.02	1.33–3.08	0.001	
Cigarettes/day smoked by family at home ^d^ ^,^ ^c^								0.589								<0.001
Never exposed	270	50.4	289	54.4	1.00				112	41.5	159	57.0	1.00			
1–4	102	19.0	89	16.8	1.16	0.81–1.65	0.410		38	14.1	30	10.7	1.81	1.02–3.22	0.043	
5–15	77	14.4	74	13.9	1.00	0.67–1.48	0.994		38	14.1	42	15.1	1.26	0.72–2.18	0.417	
≥ 16	87	16.2	79	14.9	1.13	0.78–1.65	0.510		82	30.3	48	17.2	2.57	1.61–4.09	<0.001	
Total pack-years of exposure at home ^d^ ^,^ ^c^								0.649								0.001
Never exposed	270	51.5	289	55.8	1.00				112	43.6	159	58.0	1.00			
1–4	116	22.2	97	18.7	1.18	0.84–1.67	0.339		32	12.5	27	9.9	1.65	0.89–3.04	0.109	
5–15	76	14.5	78	15.1	0.94	0.64–1.39	0.775		34	13.2	36	13.1	1.30	0.74–2.30	0.360	
≥ 16	62	11.8	54	10.4	1.17	0.76–1.80	0.479		79	30.7	52	19.0	2.34	1.47–3.74	<0.001	
Passive smoking exposure in the workplace ^e^ ^,^ ^c^																
Never exposed	196	63.6	241	74.6	1.00				75	68.2	115	81.0	1.00			
Ever exposed	112	36.4	82	25.4	1.07	0.67–1.70	0.784		35	31.8	27	19.0	1.70	0.70–4.08	0.239	
Smoker-years of exposure in the workplace ^e^ ^,^ ^c^								0.604								0.305
Never exposed	196	65.8	241	76.8	1.00				75	69.4	15	37.5	1.00			
1–15	44	14.7	34	10.8	1.17	0.64–2.13	0.609		6	5.6	6	15.0	1.18	0.31–4.44	0.810	
16–35	33	11.1	19	6.0	1.29	0.64–2.61	0.470		7	6.5	4	10.0	2.58	0.65–10.27	0.179	
≥ 36	25	8.4	20	6.4	1.11	0.53–2.31	0.789		20	18.5	15	37.5	1.45	0.39–5.33	0.575	

Abbreviation: Freq. = frequency; OR = odds ratio; CI = confidence interval.

^a^ ORs adjusted for age, residence, study stage, BMI, physical activity, age at menarche, age at first live birth, age at menopause, mother/sister/daughter with breast cancer and history of benign breast disease.

^b^ Passive smoking exposure at home or in the workplace.

^c^ Missing data were omitted from the calculation.

^d^ Excluded all women who reported only passive smoke exposure in the workplace and the ORs adjusted for age, BMI, physical activity, age at menarche, age at first live birth, age at menopause, mother/sister/daughter with breast cancer, history of benign breast disease and workplace passive smoking.

^e^ Among women who had ever been employed and excluding all women who reported only passive smoke exposure at home and the ORs adjusted for age, BMI, physical activity, age at menarche, age at first live birth, age at menopause, mother/sister/daughter with breast cancer, history of benign breast disease and passive smoking at home

We also evaluated the relationship between passive smoking and breast cancer risk stratified by ER and PR status (Table 6), excluding 92 (10.5%) cases with no ER/PR information. These analyses included 567 ER+ cases, 218 ER- cases, 596 PR+ cases and 189 PR- cases. A positive association was observed in all subtypes of ER and PR status, although the associations among women with ER+/PR+, and ER+/PR- or ER-/PR+ breast cancer tumors were not statistically significant.

**Table 6:** Association between passive smoking exposure and breast cancer risk by ER/PR status.

		ER+/PR+			ER+/PR- or ER-/PR+			ER-/PR-		
	Controls (N = 890)	Cases (N = 519)	OR (95% CI) ^a^	*P*-value	Cases (N = 125)	OR (95% CI) ^a^	*P*-value	Cases (N = 141)	OR (95% CI) ^a^	*P*-value
Passive smoking exposure										
Never exposed	448 (50.3)	243 (46.8)	1.00		54 (43.2)	1.00		52 (36.9)	1.00	
Ever exposed ^b^	442 (49.7)	276 (53.2)	1.22 (0.97–1.54)	0.081	71 (56.8)	1.40 (0.94–2.08)	0.094	89 (63.1)	1.71 (1.16–2.51)	0.006
Total smoker-years of any exposure ^c^										
Never exposed	448 (52.4)	243 (49.2)	1.00		54 (46.6)	1.00		52 (37.7)	1.00	
1–16	134 (15.7)	65 (13.2)	0.91 (0.64–1.29)	0.587	13 (11.2)	0.98 (0.50–1.93)	0.958	18 (13.0)	0.97 (0.53–1.79)	0.928
17–30	174 (20.4)	100 (20.2)	1.08 (0.79–1.47)	0.618	31 (26.7)	1.40 (0.85–2.32)	0.187	38 (27.5)	1.93 (1.19–3.13)	0.007
> = 31	99 (11.6)	86 (17.4)	1.96 (1.37–2.80)	<0.001	18 (15.5)	1.71 (0.93–3.15)	0.084	30 (21.7)	2.48 (1.44–4.24)	0.001
*P*_trend_-value			0.003			0.057			<0.001	

Abbreviation: ER = estrogen receptor; PR = progesterone receptor; OR = odds ratio; CI = confidence interval.

^a^ ORs adjusted for age, residence, study stage, BMI, physical activity, age at menarche, age at first live birth, age at menopause, mother/sister/daughter with breast cancer and history of benign breast disease.

^b^ Passive smoking exposure at home or in the workplace.

^c^ Missing data were omitted from the calculation.

## Discussion

This study found that passive smoking exposure was associated with an increased risk of breast cancer. The significant dose-response relationship between total smoker-years and breast cancer risk was found in both the pre and post-menopausal women, and in all ER/PR subtypes of breast cancer. This study also showed that passive smoking exposure at home was associated with increased risk of breast cancer, and there were significant dose-response relationships in smoker-years, cigarettes/day and total pack-years. However, no evidence of a relationship between passive smoking exposure in the workplace and breast cancer risk was found.

Results from previous studies on passive smoking and breast cancer risk were inconclusive. Some studies suggested an increased risk with passive smoking exposure [1, 9, 14–19] and others showed no effect [4, 10–13]. A meta-analysis containing seven cohort studies and twelve case-control studies supported the notion that passive smoking exposure was positively associated with breast cancer risk (relative risk = 1.27, 95% CI = 1.11–1.45) [1]. A later meta-analysis reported the combined relative risk of breast cancer in the 17 studies with retrospective reporting of exposure was 1.21 (1.11–1.32), based on a total of 5696 breast cancer women [31]. The result of the present study is consistent with that reported from the meta-analyses, showing a positive association between passive smoking and an increased risk of breast cancer.

The observed association between passive exposure to tobacco smoke and breast cancer risk is biologically plausible. Tobacco smoke contains over a dozen fat-soluble compounds that are known to induce mammary tumors in rodents [32]. Studies have strongly suggested that breast tissue is a target for the carcinogenic effects of tobacco smoke [33]. DNA adducts with derivatives of tobacco smoke are more common in the breast tissue of smokers than that of non-smokers [34–36]. It has been demonstrated that most of the tobacco smoke is not inhaled by the smokers and the highest amounts of many components, such as carbon monoxide, nicotine, benzene, formaldehyde, N-nitrosamines, nickel and tar, are found in side-stream smoke [20]. Moreover, the vapor-phase constituents from side-stream smoke are also more quickly absorbed into blood and lymph systems than the particulate-phase particulates found in main stream smoke [37].

This study found a significant dose-response relationship between total smoker-years of any exposure and breast cancer risk. Some previous studies have reported increased breast cancer risks for various duration of exposure [9, 15–17, 28], or regardless of duration of exposure [17, 38, 39]. However, many studies have not observed any linear dose–response relationship between passive smoking and breast cancer risk [4, 9, 11, 19, 38]. In this study, every smoker around passive smokers were considered and smoker-years was used. This may reflect a better measure to evaluate the duration and intensity of passive smoke exposure than years.

Inconsistencies in exposure assessment methods may contribute to the inconsistent findings across studies [18]. Ideally, an exhaustive assessment of exposure to passive smoking should include the duration and intensity of childhood home exposure, adult home exposure, and workplace exposure [27]. However, some passive smoking studies relied on husband's smoking history as the index of exposure and did not quantify additional sources of exposure [1, 4, 28, 40]. This may lead to possibly non-differential misclassification of the exposure status and may dilute the risk estimates [1]. In the present study, we had no information on childhood home exposure. Although more recent studies considered childhood exposure, almost universally, these studies tend to report null results for breast cancer risk [21]. This may in part be due to the fact that self-report of parental smoking is subject to even greater error.

Analyses according to sources of passive smoking exposure were also conducted. A positive association and significant dose-response relationships in smoker-years, cigarettes/day and total pack-years were found. Our findings are consistent with that observed in some home exposure studies [27, 28]. However, a number of home exposure studies found null association [9, 12, 13, 18, 19, 40–42]. Some [19, 40, 42] of these studies solely relied on husband's smoking history as the index of home exposure, and most [13, 40–42] studies had no information on workplace exposure. As such, inadequate passive smoke exposure assessment (for example, ignoring occupational exposure) could result in classifying those with only workplace exposure as “unexposed”, thus leading to underestimates of risks, if they should exist [20]. In the present study, we collected detailed information on home and workplace passive smoke exposure, and used no any exposure as the referent group.

We observed no significant association between workplace exposure to passive smoking and risk of breast cancer. Our results are in accordance with most other previous results [9, 12, 15, 27]. However, a case-control study conducted in Shanghai, China, found some evidence of a slightly elevated breast cancer risk associated with workplace exposure of 5 hr. or more per day (OR = 1.6, 95% CI = 1.0–2.4; *P*_trend_ = 0.02) among women who worked during the 5 years after excluding the influence of home exposure [19].

A cohort study conducted among California teachers observed an increased risk in the most highly exposed subgroup of postmenopausal women exposed in adulthood (age ≥20 years) (hazard ratio = 1.25, 95%CI = 1.01–1.56) [18]. Another cohort study observed a 32% excess risk of breast cancer associated with the most extensive exposure to passive smoking among postmenopausal women who had never been active smokers [9]. Other epidemiological studies also showed a statistically significant positive association between passive smoking and breast cancer risk in postmenopausal women [17, 19, 39, 43]. Consistent with these results, our study also provided strong supporting evidence that passive smoking was associated with an increased risk of breast cancer in postmenopausal women. One possible explanation for the positive association of passive smoking with breast cancer in the postmenopausal only might be related to the anti-estrogenic effects of passive smoking [44]. Smoking women have an earlier menopause and thus fewer years of menstruation. And cigarette smoking alters estrogen metabolism [45,46], which may contribute to the absence of a positive association of passive smoking with premenopausal breast cancer. However, some reports suggested that passive smoking was associated with an increased risk of breast cancer among premenopausal women [20, 47, 48] or both pre/post-menopausal women [27]. Since this was a stratified analysis, chance findings might arise. More studies with a larger sample size might be needed to confirm this association.

Some studies have examined the association between passive smoking and breast cancer risk by ER/PR status, and yielded inconsistent results [9, 43, 49–51]. Tong *et al*. [49] reported that passive smoking exposure from partners was associated with increased risk of ER+/PR+ breast cancer among non-smoking Chinese urban women. Morabia *et al* [50] found that passive smoking increased the risk of both ER+ and ER- breast cancer. However, other studies [9, 43, 51] found no significant association between passive smoking and breast cancer risk stratified by ER/PR status. Our study found a strong positive association between passive smoking and all subtypes of ER/PR status of breast cancer, although the association was statistically non-significant for some subtypes.

### Strengths

The present study has some strengths. We conducted detailed comprehensive measurements of passive smoking exposure at home and in the workplace, including duration and intensity of exposure (e.g., smoker-years, cigarettes/day and pack-years).The data were collected using face-to-face interviews by trained interviewers and the response rates of cases and controls were relatively high. Furthermore, some major potential confounding factors were adjusted in all logistic regression models.

### Limitations

This study had some limitations. First, selection bias was inevitable in hospital-based case-control studies. To minimize this bias, great attempt was made to recruit controls from patients with a wide spectrum of non-neoplastic conditions. Moreover, the high participation rate (96.9% and 97.1% for cases and controls, respectively) and high comparability in socio-demographic factors between the two groups indicated that selection bias should not be a serious problem. Second, recall bias was also of concern in case-control studies. To reduce recall bias, we tried to interview patients as soon as diagnosis was made and take great effort to interview cases before their surgery. Third, limited sample size in some subgroups might lead to limited power to detect the associations. Fourth, there were some missing data on the duration or intensity of exposure, which may lead to an under estimation of the association. But the percentage of missing data was less than 5%. Fifth, this study had no information on genetic polymorphisms, which had been reported to modify the association of passive smoking with breast cancer risk [20].

## Conclusions

In summary, this study suggested that passive smoking was associated with an increased risk of breast cancer among non-smoking Chinese women. A stronger positive association with breast cancer was seen among postmenopausal women and all subtypes of ER/PR status of breast cancer. Future studies are needed to confirm these results.

## Supporting Information

S1 Table (SAV)
